# Indo-Gangetic Plain experiences higher aerosol pollution than North China Plain despite lower emissions

**DOI:** 10.1007/s11356-026-38305-7

**Published:** 2026-10-19

**Authors:** Maya Nolles, Tabish Ansari

**Affiliations:** 1 Campus Fryslân, University of Groningen, Leeuwarden, the Netherlands; 2 The Netherlands Organisation for Applied Scientific Research, Utrecht, the Netherlands; 3 Air Quality Modeling for Policy Advice, Research Institute for Sustainability (RIFS) at the GFZ Helmholtz Centre for Geosciences, Berliner Strasse 130, Potsdam, 14467 Germany

**Keywords:** Air pollution, Air quality, Aerosols, PM2.5, Atmospheric science, Air pollution meteorology, Indo-Gangetic Plain, North China Plain

## Abstract

We investigate the spatiotemporal dynamics of aerosol pollution in the Indo-Gangetic Plain (IGP) and the North China Plain (NCP), two of the world’s most populated regions, against their anthropogenic emissions. Utilizing CAMS EAC4 reanalysis, CAMS-GLOB-ANT emission inventories, and ERA5 meteorological data for the year 2019, we analyze the seasonally changing relationship between PM-forming emissions and ambient PM_2.5_ concentrations. We find a significant disparity: despite the NCP emitting 65% more total anthropogenic particulates and precursors annually (28,860 KT vs. 17,464 KT), the IGP suffers higher annual average PM_2.5_ concentrations (123.6 vs. 113.2 µg/m3). Temporal and episode analyses reveal that large swathes of both regions are engulfed in polluted air during much of the year, with some respite during the summer and monsoon seasons. The NCP, with lower annual average PM_2.5_, records more total “bad air quality” days (251 vs. 238), while the IGP is characterized by fewer but more prolonged episodes, with extreme stagnation events in the Delhi region lasting up to 170 consecutive days. To quantify the regional contrast in atmospheric response, we introduce the “Pollution Forming Efficiency” (PFE) metric. Our results show that during winter, the IGP’s PFE is nearly double that of the NCP, meaning that the IGP atmosphere converts unit emissions into ambient concentrations far more effectively. We identify regional meteorology as the primary driver; the IGP is characterized by stagnant winds (< 6 km/h) and a shallow planetary boundary layer (< 300 m) leading to a consistently lower ventilation coefficient that inhibits vertical mixing and dispersion of pollution. Ultimately, our findings reveal that there is an inherent meteorological vulnerability to the IGP, and therefore, any economic growth strategy in this region must account for its limited carrying capacity. Recognizing that the IGP atmosphere uniquely amplifies the health impact of every kilogram emitted is the first step toward securing a breathable future for the more than one billion people who call these plains home.

**Supplementary Information:**

The online version contains supplementary material available at https://doi.org/10.1007/s11356-026-38305-7.

## Introduction and motivation

The Indo-Gangetic Plain (IGP) and the North China Plain (NCP) are among the world’s most densely populated regions and together home to over one billion people (Mogno et al. 2021; Li 2024). This dense concentration of human settlement—historically sustained by high levels of agricultural productivity in both regions—has given rise to high emissions of industrial and vehicular pollutants in the modern era, making these regions two of the most polluted hotspots on the planet and adversely impacting the health of these immense populations (Ramachandran and Rupakheti 2021; Lu et al. 2021; Pal et al. 2009; Wang et al. 2020; Wen et al. 2020).

Of particular concern in both the regions is fine particulate matter, or PM_2.5_, defined as particles with an aerodynamic diameter of 2.5 µm or smaller. PM_2.5_ has a large enough surface area to carry toxic substances yet small enough to bypass nasal filtration and penetrate deep into the respiratory tract (Chen and Hoek 2020). These particles can deposit in the alveolar region, causing damage through lung air exchange, posing significant health risks (Schlesinger 2007; Xing et al. 2016; Sturm 2020). Epidemiological studies have shown that chronic exposure to PM_2.5_ shortens life expectancy and increases mortality risks, as well as the incidence of asthma, respiratory, and cardiovascular diseases, which adversely affect families and put a burden on the broader economy through reduced productivity and increased healthcare costs (Pope and Dockery 2006; Yang et al. 2019). Outdoor pollution has been directly linked to increased mortality; the The State of Global Air report (Health Effects Institute, 2020) attributes 4.14 million premature deaths in 2019 to exposure to ambient PM_2.5_. Therefore, the World Health Organization (WHO) as well as national governments have prescribed safe daily and annual limits for PM_2.5_ concentrations (World Health Organization, 2021; Ministry of Environmental Protection of China and General Administration of Quality Supervision, Inspection and Quarantine, 2012; Central Pollution Control Board [CPCB], 2009).

Chemically, PM_2.5_ is not a single pollutant, but rather a composite of various chemical species. It is composed of both direct particulate emissions such as those of organic carbon (OC) and black carbon (BC) and chemically produced particles through the emissions of gaseous species such as nitrogen oxides (NO_x_), ammonia (NH_3_), sulfur dioxide (SO_2_), and non-methane volatile organic compounds (NMVOC) which can undergo gas-to-particle transformation under specific meteorological and thermodynamic conditions (Pankow 1994; Odum et al. 1996; Nenes et al. 1998; Jacobson 2005; Kroll and Seinfeld 2008; Hallquist et al. 2009; Seinfeld and Pandis 2016).

In recent decades, both India and China have undergone significant urbanization and industrialization leading to increase in PM-relevant (primary and secondary) emissions (Crippa et al. 2023, 2018; Soulie et al. 2024; McDuffie et al. 2020; Kurokawa and Ohara 2020; Wu et al. 2024; Guizzardi et al. 2025). This high-emission challenge is particularly acute in the NCP and the IGP due to the high human exposure which leads to significant health burdens. While both regions suffer from severe air pollution, their primary emission sources differ, reflecting their different stages of economic development. The NCP, which includes the provinces of Beijing, Tianjin, Hebei, Henan, Shandong, Jiangsu, and Anhui (J. Wang et al. 2019), is one of the most important social, economic, and agricultural regions in China where anthropogenic emissions are dominated by the industry, power plants, transportation, and residential coal combustion (Han et al. 2015; Zheng et al. 2018). The IGP, covering the Indian states of Assam, Bihar, Chandigarh, Haryana, Jharkhand, NCT of Delhi, Punjab, Uttar Pradesh, and West Bengal, has a different emissions profile characterized by its intensive agricultural sector (Mogno et al. 2021; Jain and Sinha 2003) including crop residue burning, transportation, residential and unorganized combustion, and industry (Mogno et al. 2021; Jain et al. 2021).

These differing regional profiles are reflected in both the direct as well as the precursor gas emissions in these regions. Previous studies have investigated these contrasts; for example, Pawar et al. (2021) found that IGP has much higher column-integrated NH3 than those for NCP despite comparable emissions suggesting a higher rate of ammonium aerosol formation in the NCP. Li et al. (2017) studied SO_2_ variability and observed contrasting SO_2_ trends in the two countries, with a decline in China and a rise in India since 2007, where India is overtaking China as the world’s largest emitter of anthropogenic SO_2_. NO_x_ emissions over East Asia (mainly driven by Chinese emissions) peaked at around 2011, while those in South Asia (mainly driven by Indian emissions) continue to rise although they remain smaller than the East Asian NO_x_ emissions (Crippa et al. 2024; Guizzardi et al. 2025). Anthropogenic NMVOC emissions continue to rise in both regions, and the Chinese NMVOC emissions are nearly double (~ 28 Tg/yr) than the Indian NMVOC emissions (~ 14 Tg/yr) (Kurokawa and Ohara 2020; Soulie et al. 2024; Crippa et al. 2024).

Various studies have previously investigated the spatiotemporal characteristics and drivers of aerosol pollution in the NCP and IGP separately. For the NCP, studies reveal significant spatial heterogeneity and higher PM_2.5_ concentrations in winter due to seasonal meteorological conditions (Hu et al. 2014; Ansari et al. 2019, 2021; Ma et al. 2023). Yao et al. (2015) found that inorganic aerosol dominated PM_2.5_ mass throughout the year but organic aerosol increased during winter. Li et al. (2020) linked high levels of carbonaceous aerosols during autumn harvests to straw crushing and biomass burning. Zhang et al. (2021) emphasized the increasing role of NO_3_^−^ in secondary inorganic aerosol formation, with rising sulfur and nitrogen oxidation ratios from 2012 to 2017. The changing contributions of different emission sectors to PM_2.5_ pollution have also been studied, with Ma et al. (2018) reporting a 34% decrease in SO_2_ emissions largely due to controls in the power sector, contrasted by a 53% increase in NO_x_ emissions driven by the industrial and transportation sectors between 2006 and 2014. Zhao et al. (2020) identified residential coal combustion as the dominant PM_2.5_ source (~ 40.5%) in rural areas during winter. Song et al. (2022) found that severe haze events in southern NCP result from a combination of local emissions and secondary pollutants, with primary and secondary SO_4_ − contributing nearly equally. Ma et al. (2021) performed a regional modelling study for two contrasting pollution episodes over NCP with several perturbations to key meteorological parameters such as boundary layer height, wind speed, temperature, and moisture and found that PM_2.5_ concentrations increase with increases in temperature and moisture but decrease with increases in wind speed and boundary layer height. Additionally, Jin et al. (2022), through a modelling study, found the NCP to be an effective PM_2.5_ contributor to regional transport to nearby regions in East Asia via a net upward transport flux from the boundary layer into the free troposphere.

For the IGP, previous studies have extensively documented spatiotemporal variability and drivers of PM, revealing that the middle and lower IGP experience the highest PM_2.5_ concentrations, with annual means exceeding 110 µg/m3 and winter peaks surpassing 170 µg/m3 (Mhawish et al. 2020). Goel et al. (2023) confirm these findings identifying highest pollution levels over middle IGP and Delhi, and emphasized the need for targeted abatement strategies. Ravindra et al. (2021) studied the drivers of PM and found that carbonaceous aerosols contribute ~ 38% of PM_2.5_ mass at an urban site in Chandigarh in the northern Indian province of Punjab with solid biomass burning and stubble burning as major sources of organic carbon during post-monsoon and winter while vehicular emissions remain significant year-round. There have also been some recent studies on aerosol composition. For example, Singh et al. (2021) reported a dominance of high molecular weight PAHs and secondary organic carbon in urban aerosols, linking them to fossil fuel and biomass combustion. Other studies have highlighted the role of aerosol meteorology feedback mechanisms in the IGP. Rahman et al. (2020) highlighted seasonal variations, with higher PM_2.5_ levels in winter due to lower dispersion and temperature inversions. Recent studies have also highlighted—through observational analysis and numerical modeling—the important role of aerosols over the IGP in increasing the duration and intensity of fog events through aerosol radiation feedback, water uptake, and fog droplet activation (Bharali et al 2024; Arun et al. 2025, 2026).

While many studies have focused on PM_2.5_ in NCP or IGP separately, some have examined both regions together often with a climate impact assessment motivation. A host of studies particularly from Ramachandran and group in the early 2020s highlighted a developing NCP-IGP dipole pattern in terms of the aerosol optical depth (AOD) over the two regions such that the AOD over NCP increased from 2001 to 2011 and then declined while AOD over IGP steadily increased throughout their analysis period of 2001 to 2020. They also highlighted that both NCP and IGP have reduced aerosol radiative forcing due to decreases in absorbing aerosols over these two decades (Khan et al. 2019; Ramachandran et al. 2020, 2021, 2022; Ramachandran and Rupakheti 2021, 2022). While these studies inform us on the trends of aerosol optical properties and their radiative impacts in the IGP and NCP, they do not examine the relationship between PM_2.5_ surface concentrations and PM-forming emissions in the two regions in the context of their seasonal cycles from an air quality perspective. This gap is critical, as seasonal variations in emissions and meteorological conditions significantly influence PM_2.5_ levels and their health impacts.

In the past decade, China has implemented aggressive emission controls through its Air Pollution Prevention and Control Action Plan (APPCAP) (Zheng et al. 2018), while a comparable policy is yet to be fully realized in India. China’s efforts led to a 30% reduction in outdoor PM_2.5_ concentrations between 2010 and 2019, largely due to the clean air actions undertaken since 2013, particularly entailing a major shift from coal to gas and a reduction in industrial emissions through the phase out of outdated technology (Health Effects Institute 2020). This success was evident across the NCP: Beijing’s annual emissions, for example, dropped from 89.5 µg/m3 in 2013 to 58 µg/m3 by 2017 (Cheng et al. 2019), with similar reductions seen in other provinces (Zhang et al. 2020). In contrast, India’s National Clean Air Programme (NCAP), launched in 2019, has been “criticized for its lack of a legal mandate and its narrow focus on cities” (Health Effects Institute, 2020) Consequently, the program has yielded mixed results. Gopikrishnan and Kuttippurath (2025) showed that while 20 out of 28 cities studied saw PM_10_ reductions of 15–60%, eight others, such as Kolkata and Durgapur, actually saw PM_10_ concentrations increase, leading to an estimated 15–50% rise in associated mortalities. As a direct result of these divergent policy paths, recent studies have shown that the overall annual average PM_2.5_ concentrations in the IGP have recently surpassed those in the NCP (Yang et al. 2018). This warrants a thorough intercomparison of the total emissions in the two regions vis-a-vis the pollution levels.

In this study, we utilize CAMS (Copernicus Atmosphere Monitoring Service) reanalysis dataset for PM_2.5_ concentrations and CAMS global anthropogenic emission inventories to investigate the seasonal dynamics of PM_2.5_ concentrations and particle emissions across the NCP and IGP, providing a more comprehensive understanding of the temporal and spatial variability of PM_2.5_ pollution with a particular focus on the pollution forming potential of these two regions. We first systematically analyze the spatial distributions and seasonal cycles of both total anthropogenic emissions and surface PM_2.5_ concentrations over the two regions. We then investigate pollution episode characteristics, specifically their frequency and duration, and introduce a “pollution-forming efficiency” (PFE) metric. Finally, we examine the role of key meteorological drivers, particularly the boundary layer height and 10 m wind speed, to explain the observed differences in pollution levels relative to emissions in the two regions. The methodology is detailed in the “Methodology and gridded datasets used” section, followed by our main results in the “Results” section, and a discussion of the findings and their policy implications as well as future research directions in the “Conclusion, limitations and future work” section.

## Methodology and gridded datasets used

In this study, we rely on three publicly available gridded datasets: the CAMS Atmospheric Composition Reanalyses 4 (EAC4) (Inness et al. 2019; https://ads.atmosphere.copernicus.eu/datasets/cams-global-reanalysis-eac4), the CAMS Global Emission Inventory (CAMS-GLOB-ANT) (Soulie et al. 2024; https://ads.atmosphere.copernicus.eu/datasets/cams-global-emission-inventories), and the ERA5 meteorological reanalyses (Hersbach et al. 2020; https://cds.climate.copernicus.eu/datasets/reanalysis-era5-single-levels).

EAC4 provides gridded data on meteorological conditions, atmospheric gases, and aerosols. EAC4 has global coverage, a 3-hourly temporal resolution from 2003 to 2024 and a spatial resolution of 0.75°✕0.75°. CAMS emission inventory provides global gridded distributions of emissions of key trace gases and primary aerosol species across 12 emission sectors, namely, agricultural livestock, agricultural soils, agricultural waste burning, power generation, fugitives, industry, residential and commercial combustion, ships, solvents, solid waste and wastewater, off-road transportation, and road transportation (Soulie et al. 2024). This dataset has a monthly temporal resolution and spatial resolution of 0.1°✕0.1°. For this study, we utilize data on key anthropogenic pollutants, including OC, BC, NO_X_, NH_3_, SO_2_, and NMVOC as they contribute directly or indirectly to aerosol pollution (Tucker 2000; Seinfeld and Pandis 2006). The ERA5 meteorological reanalysis dataset provides estimates for a large number of atmospheric, ocean wave, and land surface parameters. This global dataset features monthly and hourly values at a spatial resolution of 0.25°✕0.25° and a temporal coverage extending from 1940 to the present day. For this study, we utilize boundary layer height and 10 m wind speed as they are the primary meteorological drivers governing the vertical mixing and horizontal dispersion of pollutants, respectively.

Several previous studies have utilized the EAC4 dataset to study various aspects of pollution in Europe, North Africa, and South America (Royé et al. 2024; Gilardi et al. 2023; Houdou et al. 2024; Clauzel et al. 2025; World Bank 2025) including IGP and NCP (Budakoti et al. 2025; Zhao et al. 2022, 2025; Bhowmik et al. 2024; Kamat et al. 2025). They, arguably, provide a superior basis for pollution analyses as compared to raw model outputs because they are already synthesized and bias-corrected with satellite and, for some variables and regions, ground-based observations. When properly analyzed, they can also provide additional broad insights into regional pollution as compared to ground observations given their wider and systematic spatial and temporal coverage. Point observations, while representing the ground truth, often represent hyper local conditions which may not be very representative of a wider region. There have also been persistent issues with data quality (Pant et al. 2018; Mogno et al. 2021; Martin et al. 2019) and integrity, especially in South Asia (Mathew 2025; The Economic Times, 2025, Yadav 2025). A few studies have attempted to evaluate the gridded CAMS reanalyses dataset against ground observations (Jin et al. 2022; Ali et al. 2022; Soni et al. 2021); however, scale differences hinder a fair comparison.

For the NCP, we include the provinces of Beijing, Tianjin, Hebei, Shandong, Henan, Anhui, and Jiangsu. For the IGP, we only include the Indian provinces of Assam, Bihar, Chandigarh, Haryana, Jharkhand, NCT of Delhi, Punjab, Uttar Pradesh, and West Bengal. We obtained the shapefiles for these provinces from the Global Administrative Data database (GADM 2024) which provides high-resolution spatial information for administrative areas worldwide.

To ensure consistency across datasets, several harmonization and transformation steps were performed. First, the administrative shapefiles are utilized to mask and extract grid cells corresponding strictly to the NCP and IGP domains across all three datasets. To align the temporal resolutions, the 3-hourly CAMS concentration data were aggregated into 24-h averages for the daily variability and threshold analysis and into monthly averages to match the temporal scale of the emission inventories for the analyses which involve both the concentrations and emissions. For spatial analysis, specifically the calculation of determining the spatial extent of pollution, we utilized the actual surface area of each grid cell derived from the dataset’s map projection to account for the variation in grid cell size across different latitudes. Furthermore, to assess the total pollution potential (as described in the “Results” section), emissions were summed across all anthropogenic sectors for both primary particulates and precursor gases. Finally, to derive the pollution forming efficiency (PFE) metric (“Pollution forming efficiency in NCP and IGP” section), the spatially averaged monthly concentrations were normalized by the total aggregated emission mass rates for the respective regions. Specific details regarding the analytical implementation of these metrics are elaborated alongside the corresponding findings in the “Results” section. All data handling, analysis, and visualization were performed using the *NCAR Command Language* v6.6.2 (NCL, 2019).

## Results

In this section, we discuss the key results of our analysis. We begin by describing the spatial distribution of the total anthropogenic emissions over NCP and IGP then present various spatiotemporal aspects of PM_2.5_ concentrations, including, for example, the number and length of bad air quality episodes as distributed across the two regions. We then relate the temporal cycle of PM_2.5_ concentrations to the temporal cycle of total anthropogenic emissions as well as primary PM emissions to obtain two different kinds of pollution forming efficiencies over both regions. We find higher pollution forming efficiency in IGP as compared to NCP based on both total as well as primary PM emissions which prompts us to further diagnose key parameters of air pollution meteorology over the two regions. These results are discussed in the “Emission distributions over NCP and IGP” section to the “Air pollution meteorology in NCP and IGP: explaining differences in pollution forming efficiencies” section.

### Emission distributions over NCP and IGP

**Fig. 1:**
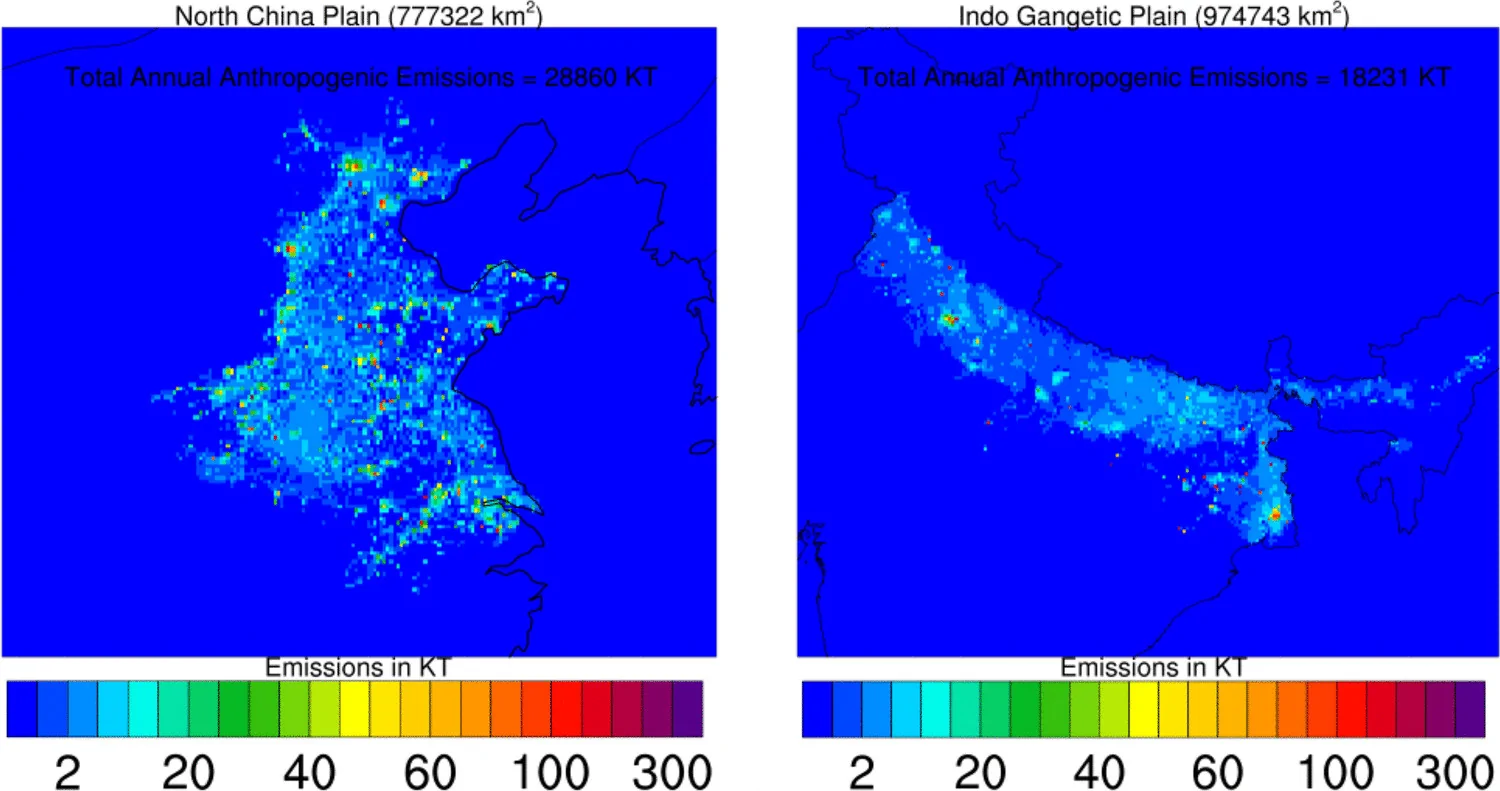
Spatial distribution of total anthropogenic emissions over NCP and IGP

Figure 1 shows the spatial picture of total anthropogenic emissions that contribute to aerosol concentrations in the North China Plain (left) and the Indo-Gangetic Plain (right) for the year 2019 as obtained from the CAMS anthropogenic emissions dataset as described in the “Methodology and gridded datasets used” section. Here, we have summed the emissions of BC and OC which form primary PM along with the emissions of gases which contribute to secondary PM formation, i.e., NH_3_, NO_X_, NMVOC, and SO_2_. Emissions from all anthropogenic sectors, including agricultural waste burning (see Methodology section), were summed to derive the grand totals shown in Fig. 1. We have not included PM-forming emissions of biogenic and natural origin such as biogenic VOCs (e.g., isoprene from trees), forest fire emissions, and natural dust. This is because we primarily focus only on emissions which can be abated through air pollution mitigation policies. We find the total PM-contributing anthropogenic emissions to be 28,860 KT for North China Plain and 17,464 KT for Indo-Gangetic Plain which are spread over a surface area of 777,322 km^2^ and 679,331 km^2^, respectively. Since we wanted to keep our analyses to a national level, we did not include provinces from Bangladesh and Pakistan which are otherwise considered part of the wider Gangetic Plains.

In terms of spatial distribution of emissions, we see large heterogeneity in both NCP and IGP. For NCP, the urban hotspots of Beijing, Tangshan, Tianjin, Shijiazhuang, Shanghai, and Changzhou clearly stand out from the rural regions with emissions upwards of 100 KT while there are pockets with negligible anthropogenic emissions, under 2 KT. We also see plenty of emission hotspots in cities dotted along the western edge of NCP along the foothills of the Taihang mountains. For IGP, we see fewer and smaller emission hotspots which are greater than 100 KT. In particular, two megacities, Delhi and Kolkata, stand out as major emission hotspots. We also see a number of smaller hotspots in the states of Punjab, Bihar, and Bengal. Another notable point is the relatively higher rural emissions in IGP—it is hard to find rural pockets in IGP with emissions as low as in some rural pockets of NCP, a feature that probably suggests a higher level of urbanization in NCP. Although, some of the low-emission pockets in NCP are due to the presence of smaller mountain ranges which are absent in the IGP. Overall, we note that the NCP is 14% larger but emits 65% more than IGP. Based on this simple macro analysis of emissions within the two regions, one may expect significantly higher PM concentrations over the NCP than the IGP. With this basic intuition and anticipation, we conduct further analyses of pollution characteristics in the two regions.

### Understanding PM_2.5_ exceedances in the two regions: spatial extent and seasonality

After establishing the basic macro picture of spatial distributions of PM-forming anthropogenic emissions over the two regions, we proceed to calculate the exceedances of daily thresholds for PM_2.5_ concentrations over the two regions. The World Health Organization (WHO) has set ambitious targets for 24 h and annual average PM_2.5_ concentrations of < 15 µgm⁻^3^ and < 5 µgm⁻^3^, respectively (World Health Organization, 2021), but these are often unrealistic to achieve in countries such as China and India which have a very high aerosol loading or in regions which have a significant presence of natural aerosols such as desert dust and sea salt (Lacey et al. 2021). Therefore, there are more realistic thresholds set by national governments to achieve in the short-to-medium term. Different countries set their own air quality indices which do not necessarily overlap with each other (Ravindra et al. 2024). For example, for China, a daily average PM_2.5_ concentration below 75 µgm⁻^3^ is considered “Good” for urban and residential regions (Ministry of Environmental Protection of China and General Administration of Quality Supervision, Inspection and Quarantine, 2012) while for India, a daily average PM_2.5_ concentration below 60 µgm⁻^3^ is considered “Satisfactory” (CPCB 2009). After carefully analyzing the national air quality guidelines for both countries, we picked two thresholds: 75 µgm⁻^3^ and 60 µgm⁻^3^ to assess the spatial extent of daily exceedances for both regions. Analyzing the pollution levels in both regions for both the thresholds allows for a like-to-like comparison of the spatial extent of pollution between the two regions. For this, we averaged the 3-hourly CAMS reanalysis data over 24 h for each grid cell within the region of interest and obtained the grid cells exceeding both these thresholds for the two regions. We finally utilized the actual grid cell area on ground for the map projection used in CAMS data to calculate the percentage area that exceeds the thresholds, for both regions.

**Fig. 2:**
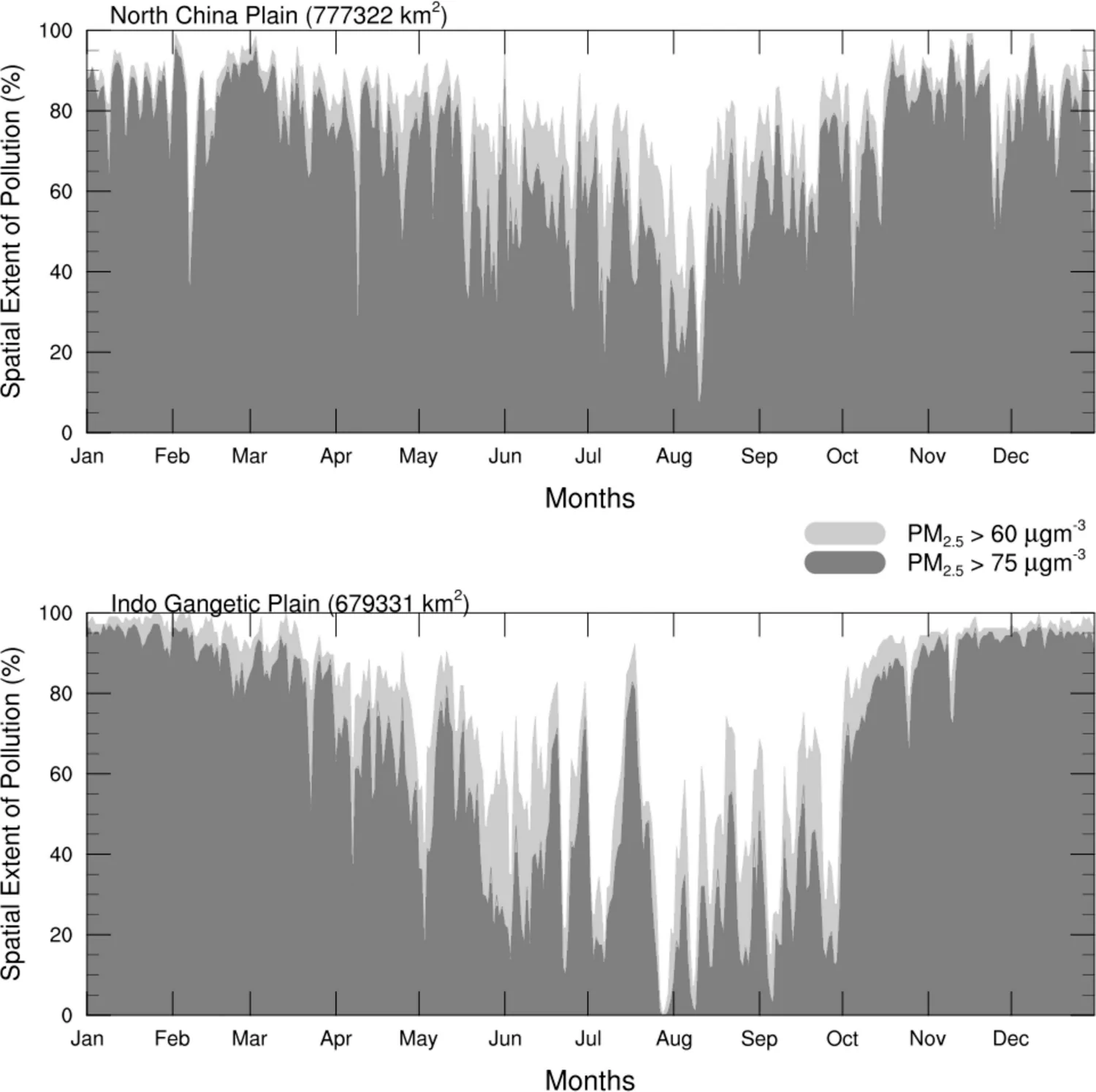
Spatial extent of daily pollution in North China Plain and Indo-Gangetic Plain for 2019

Figure 2 shows the daily time series of the spatial extent of exceedances for both regions for the year 2019. Percentage area exceeding the 75 µgm⁻^3^ daily threshold is shown in dark gray while the percentage area exceeding the 60 µgm⁻^3^ daily threshold is shown in light gray. We see a similar seasonality in both exceedances with the lower threshold exceedance leading the higher threshold exceedance. The difference between the light and dark curves denotes the area experiencing daily pollution levels between 60 and 75 µgm⁻^3^ which is small in the winter but expands in the summer and monsoon.

In winter, we see 80–90% of NCP and 95–99% of IGP exceeding the thresholds highlighting the highly large-scale regional aspect of pollution in these two important highly populated regions of the world. In such a scenario, mere city-scale pollution abatement efforts are unlikely to address the issue. This has also been highlighted by other studies such as Ravishankara et al. (2020). Crucially, large portions of low-emission zones in both regions as seen in Fig. 1 also experience poor air quality despite not contributing to high emissions, which highlights issues of environmental injustice as also noted by other studies (Do et al. 2020; Wang et al. 2022, 2023).

A large portion of NCP and IGP remains engulfed in unhealthy levels of PM_2.5_ even through spring and autumn where it gets some respite in the summer and monsoon seasons (see Fig. 2). From June to August, roughly half of NCP and 70–80% of IGP enjoys good air quality with no PM_2.5_ exceedances. Notably, on one day towards the end of July, the entire IGP experiences good air quality, with no grid cell exceeding even the lower threshold, a phenomenon not observed in the NCP. This is mainly due to higher vertical mixing in a deeper planetary boundary layer along with monsoonal convection as well as higher horizontal wind speeds. We analyze this in more detail in the “Air pollution meteorology in NCP and IGP: explaining differences in pollution forming efficiencies” section.

### Spatial distribution of PM_2.5_ concentrations and bad AQ days

**Fig. 3:**
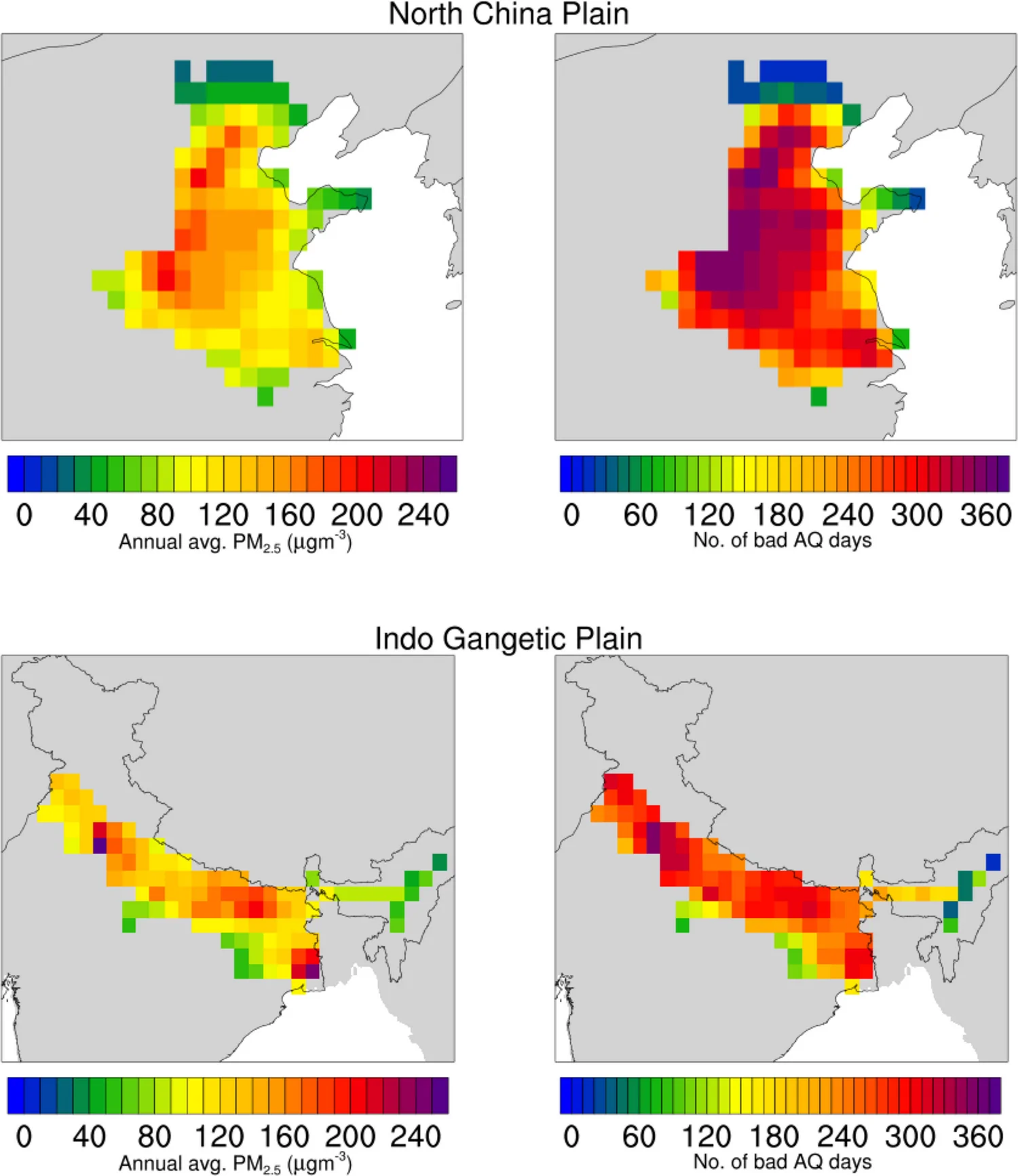
Spatial distribution of annual average PM_2.5_ and bad AQ days in North China Plain and Indo-Gangetic Plain

After getting a sense of the emission distribution and the temporal variation of air quality in the two regions, we analyze the spatial distribution of PM_2.5_ concentrations along with the geographical spread of bad air quality days in the two regions. Figure 3a and c show the annual averaged spatial distribution of PM_2.5_ concentrations for NCP and IGP, respectively. We see that both regions violate the WHO recommended limits for annual averaged PM_2.5_ (5 µg/m3) throughout their geographical area. For NCP, we see concentrations ranging from 40 to 200 µg/m3 with elevated levels from Beijing towards Shijiazhuang along the Taihang Mountains to Zhengzhou. Lower concentrations of around 30–50 µg/m3 are seen in the north, near the Yanshan mountains, and the eastern coast. The overall annual spatial average PM_2.5_ concentration for NCP is found to be 113.2 µg/m3. For IGP, we see consistently elevated concentrations in the range of 100–200 µg/m3 with particular hotspots around Delhi and Kolkata exceeding 240 µg/m3 and the greater Patna region in Bihar reaching 200 µg/m3. Lower concentrations of around 40–50 µg/m3 are only seen in the lower regions of IGP such as Bundelkhand, lower Jharkhand, and Assam. Annual spatial average PM_2.5_ concentration for IGP is found to be 123.6 µg/m3. Therefore, we see that these high values of PM_2.5_ are fairly well-distributed across the geographies of the two regions albeit the two hotspots of IGP, Delhi and Kolkata, stand out and drive the regional average up. One should note a striking point of this result: the region which emits (significantly) lower anthropogenic emissions, i.e., IGP, experiences higher annual average PM_2.5_ concentrations. This is a key result of our analyses, and we were motivated by this result to further understand the causes, characteristics, and impacts of this phenomenon.

First we begin to investigate the daily characteristics of the PM_2.5_ pollution for both regions, something which was explored in the “Understanding PM_2.5_ exceedances in the two regions: spatial extent and seasonality” section at a macro level, but here, we aim to understand the spatial detail. For this, we constructed maps for both regions showing the number of days that exceeded the 75 µg/m3 threshold for daily average PM_2.5_ concentrations. Figure 3b and d show these maps for NCP and IGP, respectively. We have already seen that most days are bad air quality days for large swathes of the two regions (Fig. 2); here, we explicitly see the geographical distribution of bad air quality days. We see that the majority of the days of the year (> 300) exceed the 75 µg/m3 daily threshold across the two regions. The average number of bad air quality days for NCP is 251 and for IGP is 238. It is interesting to note that the region with higher pollution (IGP) experiences fewer bad air quality days and vice versa. This is mainly due to the Indian summer monsoon as we will explore in further sections. Furthermore, the hotspots with high PM_2.5_ concentrations experience much more bad air quality days (~ 350). This result points to fairly uniformly occurring high pollution episodes over the year for the areas with high annual average PM_2.5_. We explore the frequencies of occurrence of these episodes in the next section.

### Episode characteristics in the two regions

We define an air quality episode as the duration over which the daily PM_2.5_ concentration continuously exceeds the daily national standard of 75 µg/m^3^. We analyze various episode characteristics for each grid cell over the two regions: the number of air quality episodes in 1 year, the average duration of an episode, and the duration of the longest episode.

**Fig. 4:**
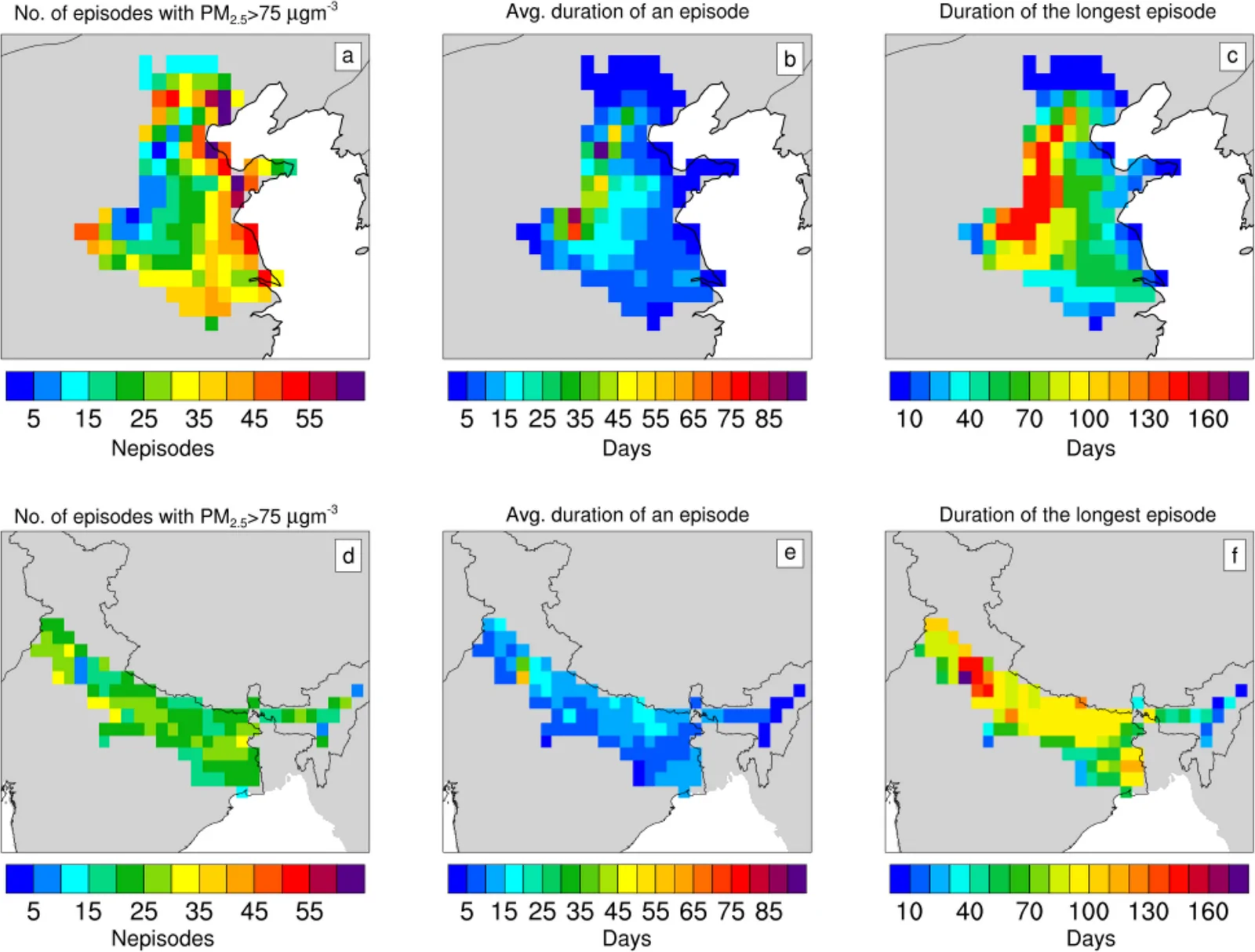
AQ episode characteristics for North China Plain and Indo-Gangetic Plain

Figures 4a and d show maps of total number of bad air quality (AQ) episodes across NCP and IGP, respectively, for the year 2019. As noted earlier, we define an episode as the total consecutive number of days with daily average PM2.5 concentrations > 75 µg/m3 until there is a day with lower PM_2.5_ concentrations. One would generally assume grid cells with higher number of AQ episodes to be more polluted; however, in highly polluted regions such as NCP and IGP which show consistent pollution, counterintuitively, higher number of AQ episodes would mean higher clean air intervals (i.e., more breaks in bad air quality). Therefore, these maps look almost the opposite of the PM_2.5_ concentration maps (see Fig. 3a and c). Here, grid cells with lower AQ episodes mean fewer but very long episodes, sometimes spanning the entire year. Based on these results, it became imperative to expose the duration of episodes per grid cell—both the average duration and the maximum duration. These are shown in the remaining panels of Fig. 4.

Figure 4b shows the average duration of AQ episodes for NCP, and here, we see very high duration episodes, 30–80 days long, in grid cells with high annual average PM_2.5_, in the western NCP. As we move towards the east, the average AQ episode becomes shorter (and more frequent if we see Fig. 4a), down to 5–15 days long. Figure 4e shows the average duration of AQ episodes for IGP. Here, we rarely see any grid cell exceeding an average duration of 20 days, except for that one grid cell representing Delhi, with an average duration of ~ 60 days. This is an interesting result suggesting that IGP, despite experiencing higher PM_2.5_ pollution than NCP, experiences it in shorter, more frequent episodes. There is also an interesting contrast between the two major high PM_2.5_ hotspots of IGP, i.e., Delhi and Kolkata: while Delhi experiences fewer but longer AQ episodes, Kolkata experiences shorter but more frequent episodes. This result can be clearly seen from the corresponding gridcells in the panels d and e. It would be valuable to investigate the reasons for these frequent breaks in the AQ episodes in Kolkata in terms of local emission characteristics and meteorology. However, a city-level analysis is beyond the scope of this study.

Finally, we also calculated the duration of the longest episode for each grid cell for both regions—these are shown in Fig. 4c and f. For NCP, we see a high resemblance of the spatial distribution of the duration of longest episode with the spatial distribution of annual average PM_2.5_ concentrations: grid cells with higher annual PM_2.5_ concentrations generally experience longer longest episodes. The polluted areas along the western NCP experience bad AQ for up to 140 days continuously, but as we move towards the east, there is more respite. For IGP, much of the region experiences at least one episode that is ~100 days long; a long wait until a break day. Delhi and its surrounding regions experience a very long longest episode, spanning around 150–170 days. This is longer than any other area in the entire NCP and IGP. The regions in the far east of IGP, in Assam, experience very short spells of bad air quality where the longest episode spans around 10 days.

### PM_2.5_ concentrations vis-a-vis total emissions: seasonal cycle

After an analysis of the episode characteristics in the two regions, we return to the striking result exposed in the “Spatial distribution of PM_2.5_ concentrations and bad AQ days” section that the region with lower anthropogenic emissions experiences higher pollution. Here, we attempt to better understand this result by simultaneously analyzing the temporal cycle of PM_2.5_ concentration alongside the temporal cycle of emissions in both regions. Also, so far, we have only analyzed the spatial picture of annual total anthropogenic emissions in the two regions (Fig. 1); however, to better understand aerosol formation, we need to understand the temporal cycle of both the primary PM emissions, i.e., OC and BC and gaseous emissions that contribute to secondary (organic and inorganic) aerosol formation, i.e., NH_3_, SO_2_, NO_x_, and NMVOC.

**Fig. 5:**
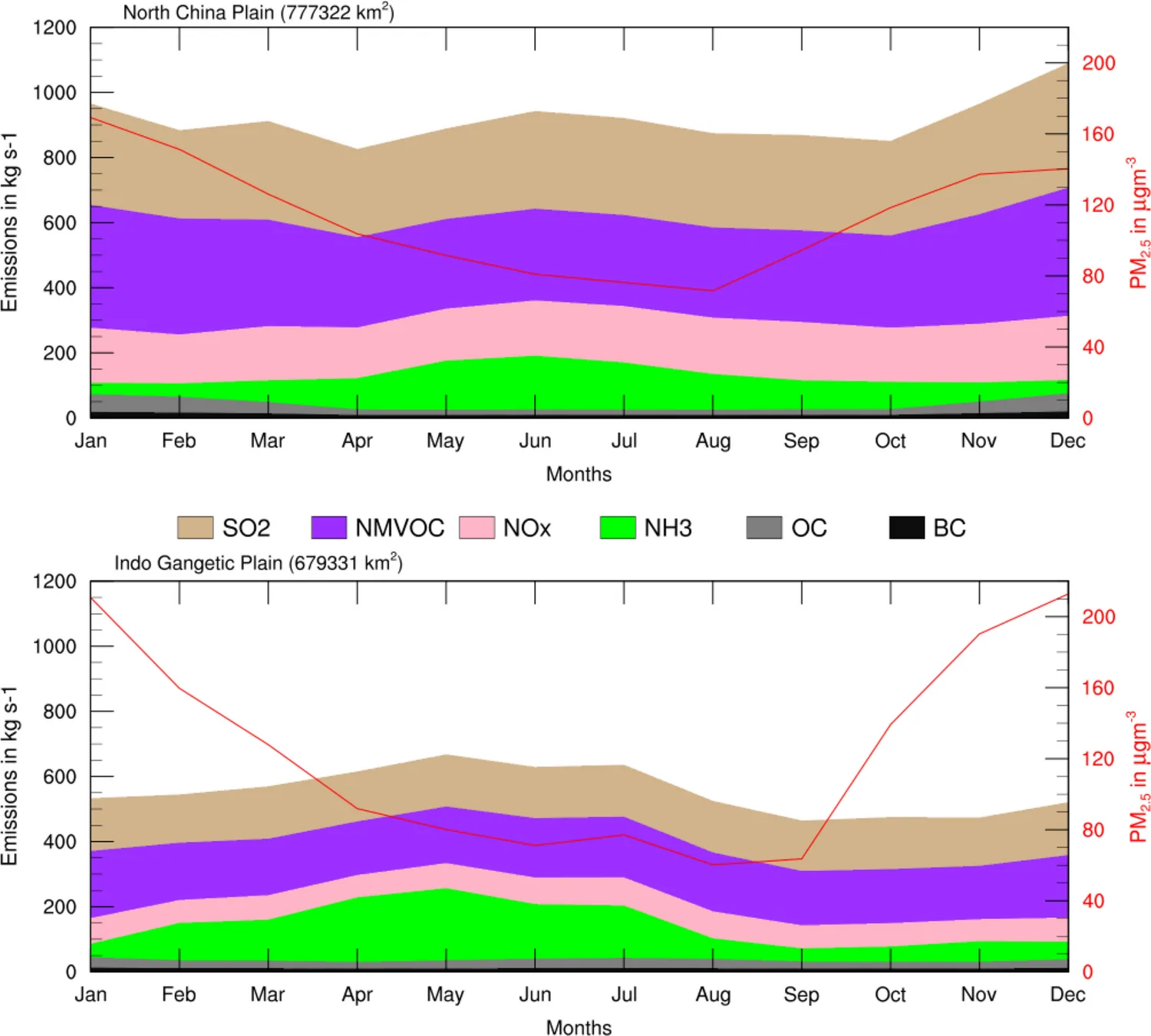
Seasonal cycle of total anthropogenic emissions versus average PM_2.5_ concentrations for NCP and IGP

Figure 5 shows the region-averaged seasonal cycle of both gaseous (NH_3_, NO_x_, NMVOC and SO_2_) and particulate emissions (OC and BC) for NCP and IGP in mass units along with the seasonal cycle of region-averaged PM_2.5_ concentrations (shown in red line). Here, we note that the gaseous emissions are much larger (95% of total emissions by mass) than the primary particulate emissions. The chemical composition of these emissions is quite different from typically observed aerosol composition in these regions, wherein the secondary aerosols, formed by gaseous emissions, do not constitute ~ 95% of PM mass. Instead, they generally remain below 50% of total PM mass, only occasionally exceeding up to 70% during some extreme episodes in high emission hotspots (e.g., Huang et al. 2014; Sun et al. 2016 etc.). This demonstrates that only a low percentage of gaseous emissions convert into the solid phase, as quantified by the sulfur and nitrogen oxidation ratios ranging between 0.1 and 0.4 in these environments (Hua et al. 2015; Seinfeld and Pandis 2016).

Another unintuitive observation is that for both regions, but particularly for IGP, the PM_2.5_ concentrations are lowest when the total contributing emissions are at their highest, i.e., in the summer, particularly driven by high NH_3_ emissions related to agricultural activity (Pawar et al. 2021; Wang et al. 2020; Zheng et al. 2018). This clearly highlights the defining role of meteorology which can disperse pollution at the surface despite high emissions—the role of Indian monsoon here cannot be understated (Fig. 2; Rajeevan et al. 2025). The PM_2.5_ concentrations in both the NCP and IGP are lowest between April and September and highest during the remaining colder months. In the NCP, concentrations peak around 140 µg/m3 in January and December, with the lowest levels, about 70 µg/m3, occurring in September. For the IGP, concentrations exceed 200 µg/m3 during winter months and are lowest at approximately 60 µg/m3 during the monsoon season. If we analyze the temporal variation of PM_2.5_ only against the primary emissions (OC and BC), we do see a more correlated behavior, particularly for NCP. Overall, it becomes clear through this analysis that the higher concentrations seen in IGP despite lower emissions as noted in the previous section "Spatial distribution of PM2.5 concentrations and bad AQ days" is really driven by the wintertime jump in the PM concentrations.

The NCP consistently emits more than IGP throughout the year and almost double (~ 1100 kg/s) that of IGP emissions (~ 550 kg/s) during winter. This significant difference points to the greater pollution-forming (or pollution-trapping) potential of the IGP region. These results led us to investigate the meteorological changes occurring from the monsoon-to-winter transition in IGP that enabled such a dramatic rise in aerosol levels. We present those results in the subsequent section "Air pollution meteorology in NCP and IGP", but first, we proceed to do a similar concentration vs emission analysis only for the primary PM emissions in the next section.

### PM_2.5_ concentrations vis-a-vis primary particulate emissions: seasonal cycle

**Fig. 6:**
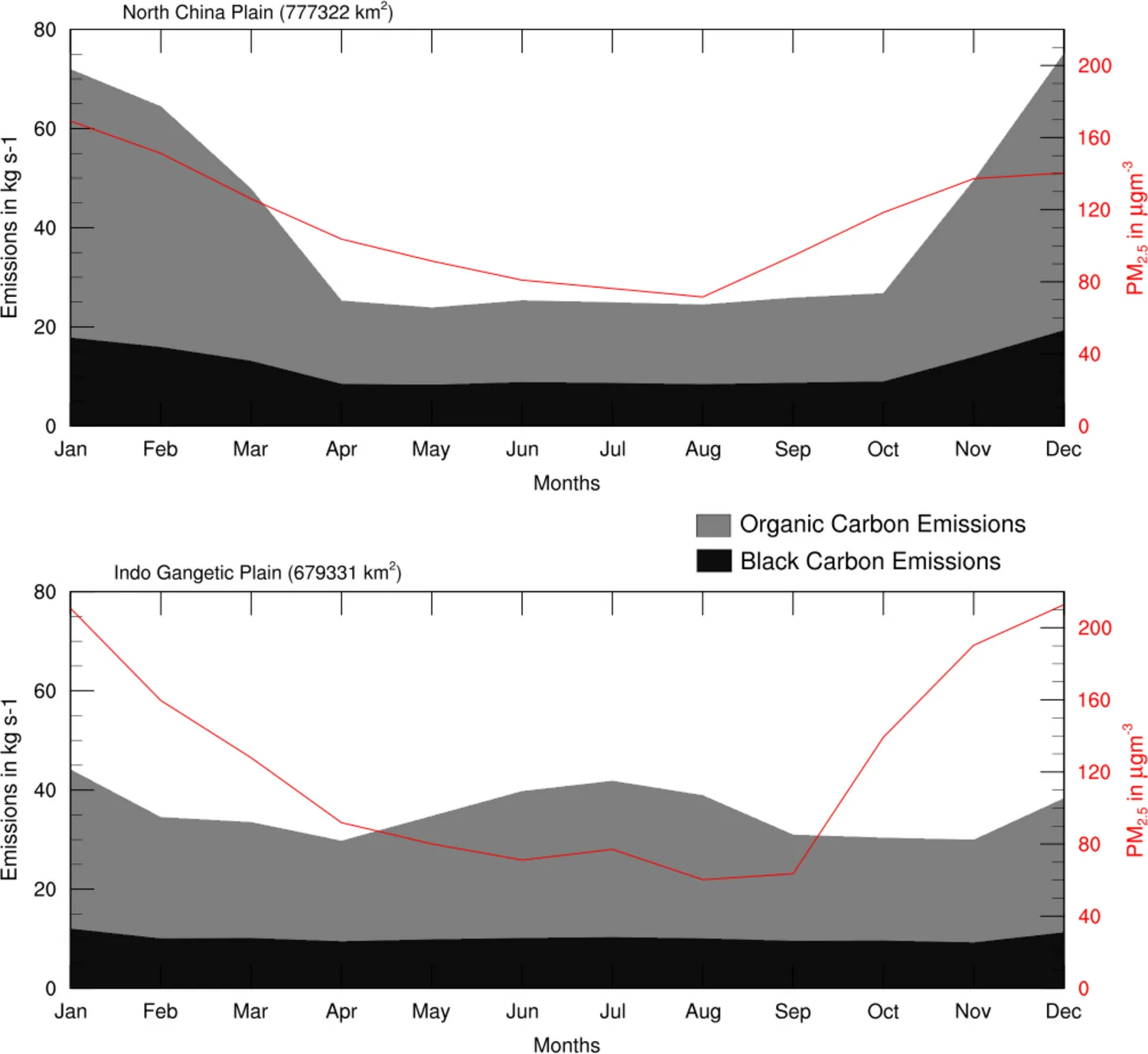
Particulate emissions versus average PM_2.5_ concentrations for NCP and IGP

Here, we present a similar temporal analysis of PM_2.5_ concentrations versus the regional emissions but without the gaseous emissions (see Fig. 6). We mainly focus on the winter months (Jan–Feb and Oct–Dec) which record the highest PM_2.5_ concentrations in both regions. We note that the primary PM emissions in NCP are higher than those in the IGP (annual average of 40.4 kg s⁻^1^ vs. 35.5 kg s⁻^1^), yet the concentrations in NCP are lower than those in IGP (annual average of 113.2 µg m^−^3 as compared to 123.6 µg m⁻3). In the spring–summer and monsoon period (April to September), IGP has higher primary PM emissions at 36 kg s⁻^1^ as compared to 24.9 kg s⁻^1^ for NCP yet experiences considerably lower PM_2.5_ concentrations (74 µg m⁻3) than NCP (86.4 µg m⁻3) during this period. This is a striking result: IGP experiences higher pollution than NCP when it emits less and lower pollution than NCP when it emits more. This result highlights the important role of the Indian summer monsoon as well as the wintertime regional meteorology in IGP in determining the PM pollution for this region (Ansari et al. 2016).

### Pollution forming efficiency in NCP and IGP

**Fig. 7:**
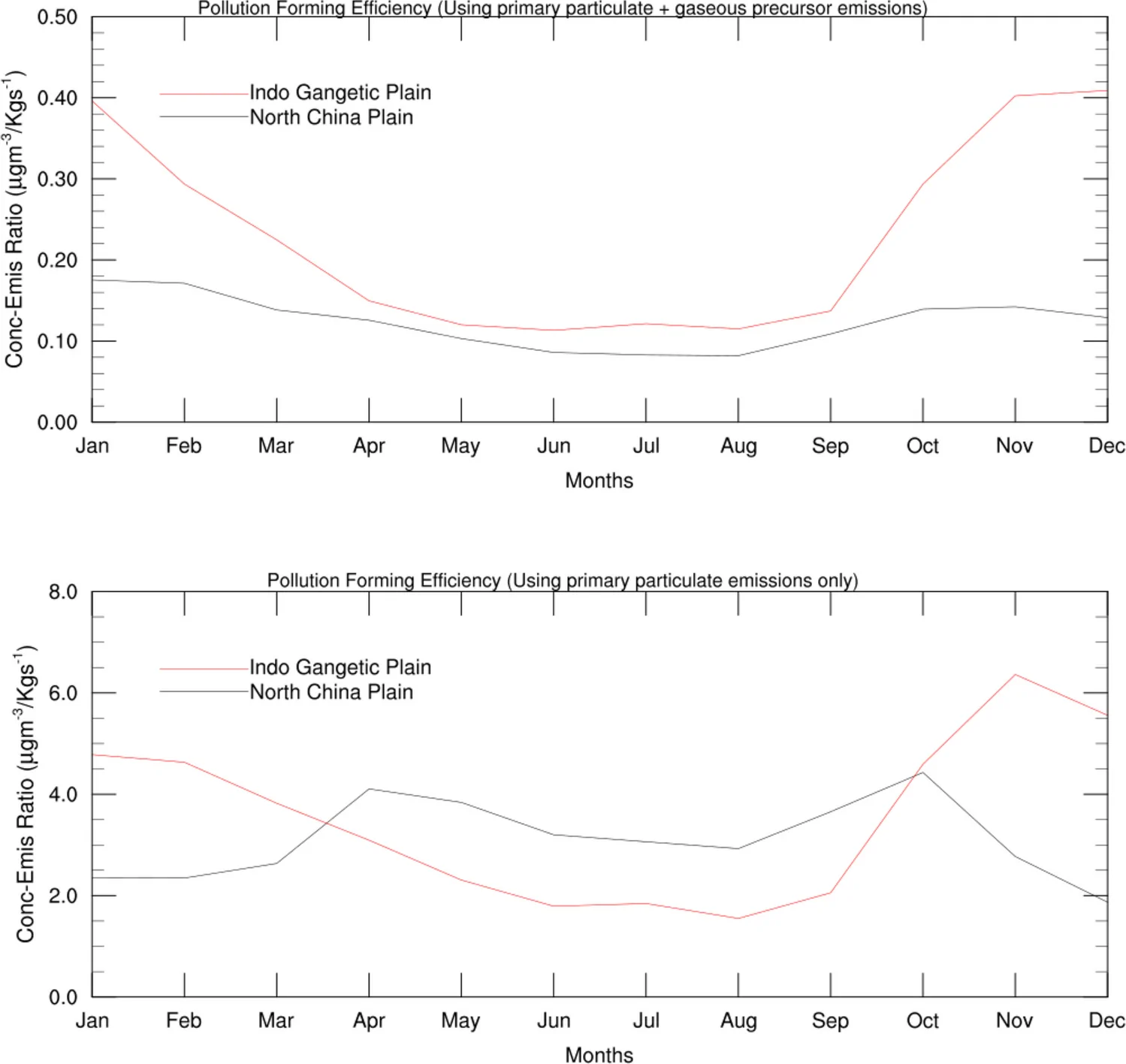
Pollution forming efficiency for North China Plain and Indo-Gangetic Plain calculated by normalizing spatially averaged PM_2.5_ concentrations by emissions

Motivated by the results of the temporal analyses of the “PM_2.5_ concentrations vis-a-vis total emissions: seasonal cycle” and “PM_2.5_ concentrations vis-a-vis primary particulate emissions: seasonal cycle” sections, we calculate the monthly pollution-forming efficiency (PFE) of the two regions through dividing the regional PM_2.5_ concentrations by (a) total emissions and (b) primary PM emissions only. We have plotted the temporal cycle of the derived PFE for the two regions in Fig. 7. In Fig. 7a, which shows a PFE based on total emissions, we see that NCP experiences a PFE of around 0.10 µgm^−3^/Kgs^−1^ in the summertime which increases up to 0.18 µgm^−3^/Kgs^−1^ in winter. IGP shows a higher PFE than NCP throughout the year, with values around 0.15 µgm^−3^/Kgs^−1^ during summertime, increasing up to 0.4 µgm^−3^/Kgs^−1^ in winter. For PFE based only on primary emissions of OC and BC (Fig. 7b), we see that both regions have a substantially higher pollution forming efficiency. This is understandable because primary emissions do not have to undergo a gas-to-particle formation process. In this case, NCP has a higher PFE during spring and summer with values around 4.0 µgm^−3^/Kgs^−1^ while IGP’s PFE drops down to 2.0 µgm^−3^/Kgs^−1^ during monsoon season. In wintertime, the primary emission PFE is at 5–6 µgm^−3^/Kgs^−1^ for IGP and 2–4 µgm^−3^/Kgs^−1^ for NCP. Crucially, in wintertime, the two regions show diverging behavior in terms of primary emission PFE, with increasing PFE for IGP and decreasing PFE for NCP.

### Air pollution meteorology in NCP and IGP: explaining differences in pollution forming efficiencies

**Fig. 8:**
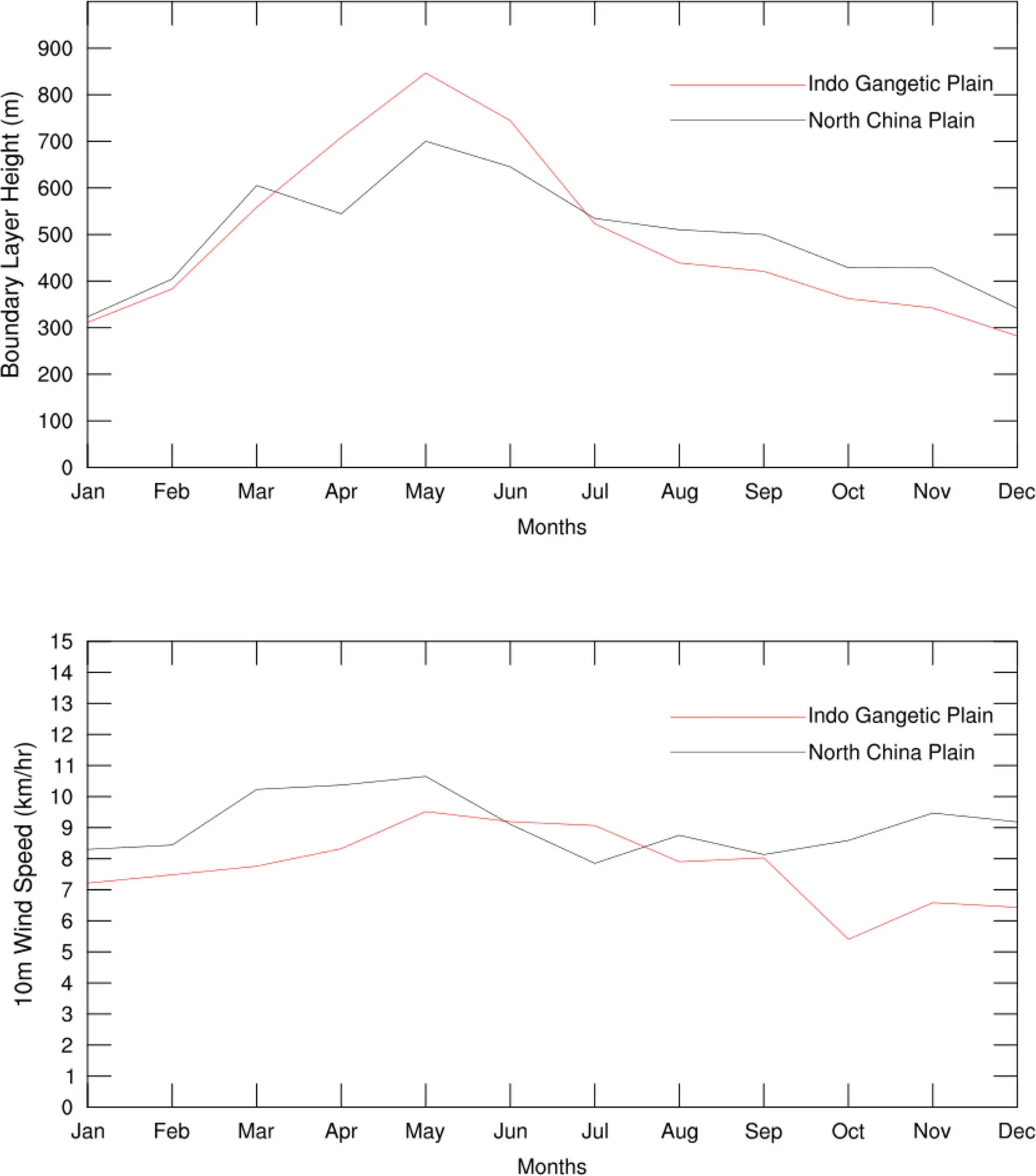
Monthly time-series of spatially averaged boundary layer height and 10 m wind speed over North China Plain and Indo-Gangetic Plain for 2019

Finally, to provide a mechanistic explanation of the results shown in the previous sections and to gain a deeper physical understanding of pollutant dispersion in NCP and IGP, we derived the region-averaged boundary layer height and 10 m wind speed using the ERA5 reanalysis dataset (Hersbach et al. 2020). Generally, from a vertical perspective, a shallower planetary boundary layer (PBL) height would trap the pollutants close to the surface thereby shooting up the surface concentrations while a higher boundary layer height would ensure more vertical mixing of pollutants leading to dilution with cleaner air thereby reducing the surface concentrations. From a horizontal dispersion perspective, lower surface wind speeds would lead to more stagnation allowing the build-up of local emissions whereas higher wind speeds would bring in cleaner air masses that would advect the polluted air from a given region. We present these meteorological characteristics for NCP and IGP in Fig. 8 which clearly explain the differences in the pollution formations in the two regions. We see substantially higher PBL height (Fig. 8a) for IGP during the summer and monsoon (600–850 m) while a lower PBL height for NCP (550–700 m). For the remainder of the year, IGP shows a consistently lower PBL height than NCP, dropping below 300 m in December, which is consistent with the dramatic rise in PM_2.5_ concentrations in IGP despite the lower emissions.

This wintertime suppression of the boundary layer in both NCP and IGP is likely exacerbated by the BC-PBL feedback mechanism, often referred to as the “dome effect” (Ding et al. 2016). In this process, absorbing aerosols like black carbon (BC) heat the upper boundary layer while reducing solar radiation at the surface, thereby increasing atmospheric stability and suppressing vertical mixing. This allows the wintertime PBL to compress, reaching monthly averages of ~ 300 m as seen in Fig. 8a, while ground-based observations have recorded collapse to even lower levels (< 200 m) during severe winter fog episodes (e.g., Tiwari et al. 2014). This compression increases local aerosol concentrations (Ding et al. 2016; Bharali et al. 2024).

IGP also experiences calmer winds than NCP almost throughout the year except the monsoon period, with 10 m wind speeds dropping down to ~ 6 km/h in post-monsoon and winter (Fig. 8b).

**Fig. 9:**
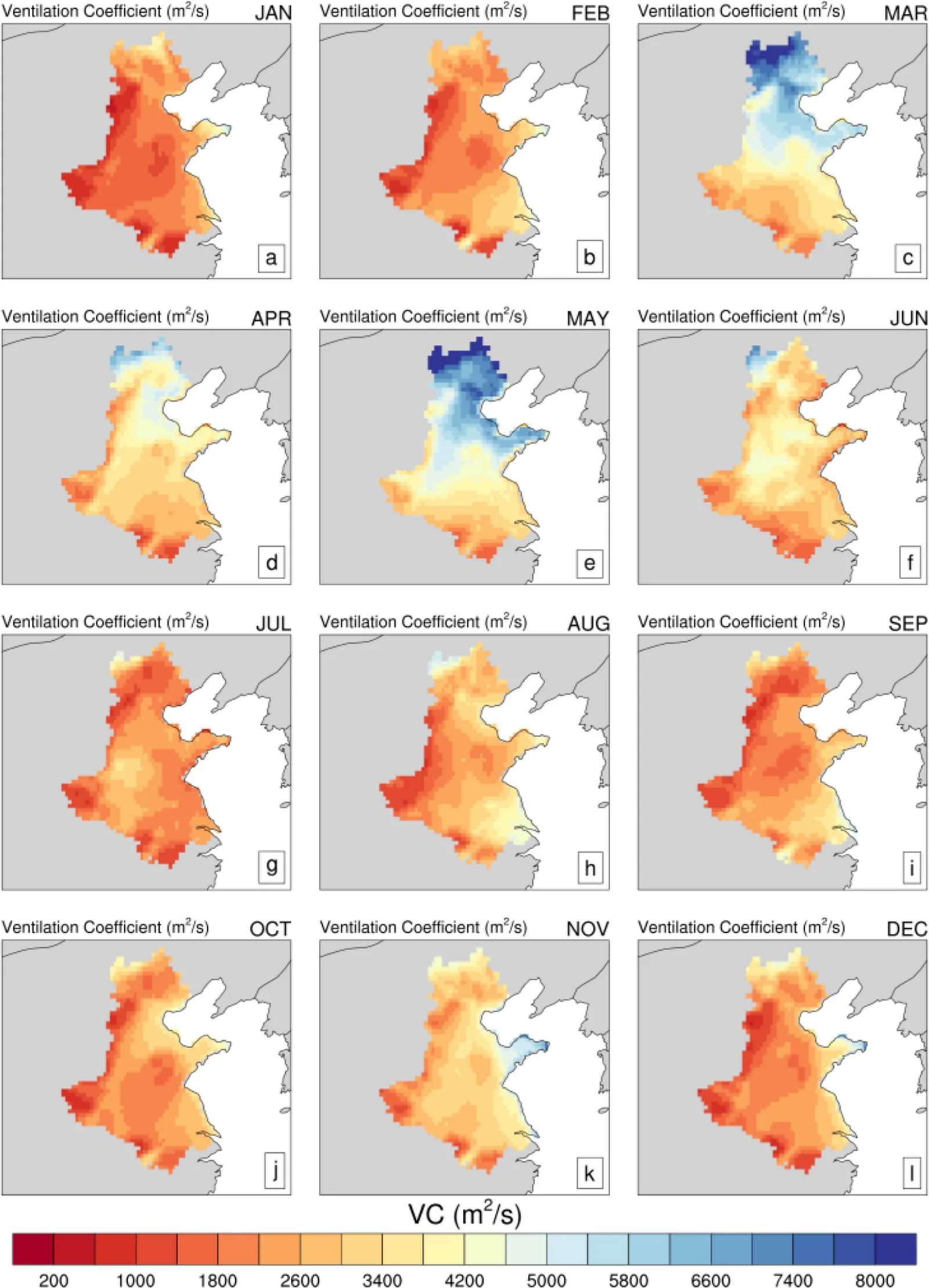
Spatial distribution of monthly ventilation coefficient over North China Plain for 2019

**Fig. 10:**
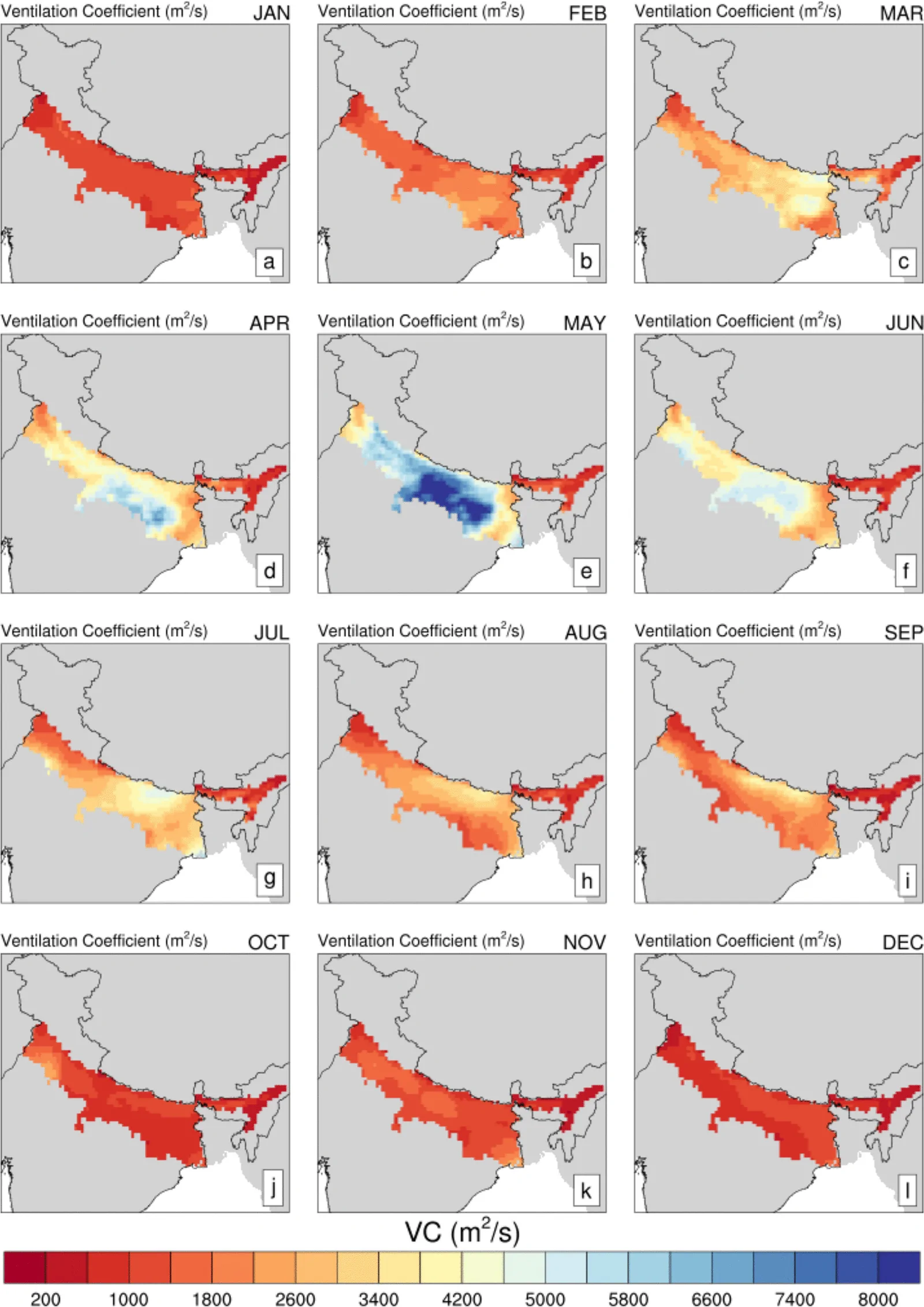
Spatial distribution of monthly ventilation coefficient over Indo-Gangetic Plain for 2019

To provide a more definitive causal mechanism, we compute the ventilation coefficient (VC) over both the study regions. Ventilation coefficient (reported in m^2^/s) is defined as the product of the boundary layer height (in m) and the vertically averaged horizontal wind speed (in m/s) up to the boundary layer height (Holzworth 1967; Krishnan and Kuhnikrishnan 2004). To compute the VC for NCP and IGP, we utilize the hourly horizontal wind speeds (reported as wind components *u* and *v*) from the ERA5 dataset available at different pressure levels. We first calculate the corresponding ERA5 vertical pressure level to the PBL height for each grid cell per hour and perform a volumetric vertical averaging of wind speeds up to this vertical level. The product of the vertically averaged wind speed and the PBL height gives the VC in m^2^/s. We present maps of monthly averaged VC for NCP and IGP in Figs. 9 and 10, respectively; the minimum, maximum, and regional mean values of monthly averaged VC for both regions are tabulated in Table 1. We found that, as compared to the NCP, the IGP experiences a significantly lower mean VC for large parts of the year (Jan–Mar and Aug–Dec) and a substantially lower minimum VC throughout the year. The NCP also consistently shows a higher local maximum VC than IGP for all months except April and July (see Table 1). For NCP, the regional mean VC ranges from 1893 to 4677 m^2^/s which is broadly consistent with VC reported in previous studies. For example, Zhu et al. (2018) found the VC derived from ceilometer observations in three cities within the NCP to be within 1000–5000 m^2^/s. Similarly, Li et al. (2022) calculated the VC over three sites within the NCP using sounding balloons to range from 1439 to 4031 m^2^/s for winter and from 3834 to 4965 m^2^/s for summer.

**Table 1:** Local minimum, local maximum and regional averaged (mean) monthly ventilation coefficients (m^2^/s) for NCP and IGP

Months	North China Plain			Indo-Gangetic Plain		
	Min	Mean	Max	Min	Mean	Max
January	448.8	1893.3	5515.0	208.3	1103.1	1721.7
February	643.0	2265.4	5059.8	317.3	1670.2	3047
March	1417.5	4348.3	8792.9	467.0	2735.5	4771
April	1137.2	3374.0	6875.3	396.8	3631.2	7093.2
May	1500.3	4677.2	11,456.2	299.6	5237.3	9308.5
June	1299.8	3162.7	7249.9	243.4	3603.5	5851.2
July	946.9	2177.2	4844.3	221.3	2721.5	5745.4
August	937.2	2674.6	5288.5	227.5	2053.8	3859.9
September	791.7	2349.4	5523.2	151.6	1897.0	3971.6
October	829.0	2333.6	4979.3	229.9	990.2	2428.3
November	1218.2	3145.3	7601.2	197.2	1194.9	2591.0
December	558.6	2281.6	7081.2	196.6	894.5	1789.7

For IGP, the regional mean VC varies between 894 and 5237 m^2^/s for 2019 (Table 1). The VC values reported from observational studies over IGP sites lie within the range of our calculated VC here. For example, Prakash et al. (2022) report an observationally derived VC over a rural site in the IGP to lie under 500 m^2^/s during high pollution episodes in autumn and winter but also increasing up to 4639 m^2^/s during the summer. Gani et al. (2019) report VC for Delhi lying within our VC range as shown in Table 1. Similarly, Ojha et al. (2020) report very low values of WRF-Chem model-derived VC (~ 400 m^2^/s) over large parts of IGP during October–December months. While previous studies have reported VC over individual sites for either NCP or IGP or only for a specific season, here, we have presented year-long monthly VC over both regions which makes the central argument of this study clear: of these two most populous regions of the world, IGP experiences a much more stagnant atmosphere which promotes more air pollution despite lower emissions.

## Conclusion, limitations and future work

In this study we have investigated the PM_2.5_ pollution characteristics in two of the world’s most populous regions, the North China Plain (NCP) and the Indo-Gangetic Plain (IGP) over a full year period. We have found that large swathes of both regions experience very high aerosol pollution far exceeding national air quality standards on most days of the year. PM_2.5_ concentrations are highest in the winter for both regions, and the few days of good or satisfactory air quality occur during the summer or monsoon period facilitated by stronger winds and vertical mixing in the boundary layer. Generally, the hotspots with the highest annual average PM_2.5_ also experience the longest air quality episodes in both regions, although with some exceptions. Crucially, we found that despite NCP generating approximately 65% more total anthropogenic aerosol precursors and primary particulates annually than the IGP (28,860 KT vs 17,464 KT), IGP experiences higher annual average PM_2.5_ concentrations (123.6 µg/m3 vs 113.2 µg/m3). During the post-monsoon and winter months, the pollution forming efficiency in the IGP effectively doubles that of the NCP, meaning the IGP atmosphere converts a unit of emission into ambient concentration far more effectively than the NCP atmosphere. We identified regional meteorology as the dominant driver of this contrast. While the NCP benefits from relatively better ventilation, the IGP is subjected to a meteorological trap characterized by calmer winds and a shallower planetary boundary layer (< 300 m) during winter which inhibits dispersion and concentrates pollutants near the surface.

We started this study during the post-covid lockdown phase when restrictions on daily socioeconomic activity were still being eased; therefore, we could not select a more recent year such as 2021 or 2022 for our analyses because emissions were still not at their baseline levels in both these regions then. Therefore, we made a pragmatic choice to select the most recent year before covid lockdowns, i.e., 2019. We recognize that emissions in both the regions are changing rapidly, decreasing in NCP and increasing in IGP, thanks to the new aggressive clean air action plan in China implemented since 2013 (APPCAP, Han et al. 2015; Zheng et al. 2018; Cheng et al. 2019; Zhang et al. 2021) and continually increasing fossil fuel-driven activity in IGP (Crippa et al. 2023; Guizzardi et al. 2025) despite some city-level emission abatement plans with partial success (NCAP; Xie et al. 2024). However, our results and conclusions are fundamental and do not depend on the absolute values of emissions in the present day. They depict the fundamental difference in the pollution forming potentials of the two regions which will remain true regardless of the changes in prevailing emissions.

Our results have strong implications for environmental and economic policy, especially for the IGP region where emissions are still increasing. Given the region’s severely limited meteorological carrying capacity, expanding heavy industry and coal-fired power generation within the IGP risks pushing an already critical public health situation into a permanent state of emergency. The Indian environmental agencies face an uphill task of reducing pollution in an unfavorable meteorological environment, particularly in the winter months (Bharali et al. 2024; Ramachandran and Rupakheti 2022). Policymakers in India must therefore carefully weigh emissions-driven economic development in the IGP against public health and well-being. Our results provide a strong incentive for a rapid transition to clean energy in northern India.

While our study relies upon widely used and accepted publicly available datasets, there is inevitably scope for biases and uncertainties. First, the CAMS anthropogenic emissions inventory could include uncertainties particularly over the IGP where local emissions datasets are still not well-developed. In particular, agricultural waste burning emissions over the provinces of Punjab and Haryana have been reported to be inconsistent across different emission datasets (Biswal et al. 2025; Ambulkar et al. 2025). This is because these emissions ultimately rely upon fire counts detected by low-Earth-orbiting satellites which have a limited overpass period when the small prevailing fires may not be representative of the overall burning activity. A recent study from the Indian Space Research Organization (ISRO) has highlighted discrepancies in crop burning trends when detected through low-Earth orbiting vs. higher geostationary satellites (Singh et al. 2025). There may also be other unaccounted emissions from informal sectors such as open waste burning and unregulated brick kilns which are indeed dotted across the IGP (Misra et al. 2020; Sharma et al. 2019). It is therefore possible that a portion of the high concentrations attributed here to meteorological stagnation could stem from unaccounted emissions. Second, the 3-hourly surface concentrations of PM_2.5_ taken from the CAMS reanalyses dataset that form the backbone of all our downstream analyses may include biases over the study regions. Generally, reanalyses data are superior to raw model output because they have been constrained or assimilated by observations. In case of CAMS reanalyses, PM_2.5_ surface concentrations are indirectly constrained through an optimization process of matching satellite-detected AOD over the region rather than directly assimilating ground-based observations, therefore still amenable to errors in vertical distribution of aerosol. However, it is not meaningful to directly compare PM_2.5_ surface concentrations to point observations on ground, given the coarse spatial resolution of the dataset (0.75°✕0.75°) and high natural spatial heterogeneity of PM_2.5_, especially in polluted regions (Taksibi and Ansari 2022; Souri et al. 2022). There has also been a persistent issue with the data quality and integrity over the IGP (Vohra et al. 2024; Mathew 2025; Yadav 2025) which prevents a direct comparison. However, since we utilize the same reanalysis dataset to assess the pollution over both regions, the systematic biases in the modeling and assimilation/optimization system are common across both regions and do not hinder the fundamental comparative conclusions. Moreover, the difference in annual averaged regional ventilation coefficients (2807 m^2^/s for NCP vs. 2311 m^2^/s for IGP) support the robustness of our conclusions despite emission uncertainties.

Our findings can benefit from further studies which could provide multiple lines of evidence on the question of comparative pollution forming potential of the NCP and IGP. A more comprehensive analysis involving multiple emission inventories, especially including bottom-up estimates, and multiple reanalysis datasets or model outputs over the two regions could further corroborate the findings. A more recent year reflecting current conditions could be chosen, or better, a multi-year study could be conducted to ascertain if the differences in pollution characteristics and pollution forming efficiencies are retained across a long time period. A more elaborate 3D analysis of the vertical distribution of aerosol in the two regions could also be performed using reanalysis products or independent model outputs. It would also be very valuable to try to separate the physical and chemical processes that drive the overall pollution forming potential in the two regions, i.e., how much of the increases in surface concentrations are due to mere physical trapping of emissions and how much extra chemical production occurs due to such trapping under stagnant conditions in both regions, and are the chemical processes key in driving up the pollution forming efficiency in the IGP? The primary PFE could also be calculated in a different way, with the numerator consisting of only the primary aerosol components for both regions if they can be made available through model simulations. Careful modelling studies with perturbed meteorological and process parameters (e.g., Wild and Ryan 2025) for example by increasing boundary layer height or mixing coefficient and wind speeds could further corroborate our results and support policy prescriptions. We have analyzed only that part of the IGP that lies within the Indian national boundaries in order to support national-level clean air policies; however, it would also be valuable to conduct a similar analysis for the wider IGP extending through Bangladesh in the east and into Pakistan in the west.

Ultimately, our findings reveal that there is an inherent meteorological vulnerability to the IGP and it needs a specialized strategy that accounts for its limited carrying capacity. This year-long analysis provides a first baseline, but moving forward, expanding this research into longer, multi-decadal studies will be vital to verify these trends against a changing climate. Recognizing that the IGP atmosphere uniquely amplifies the health impact of every kilogram emitted is the first step toward securing a breathable future for the more than one billion people who call these plains home.

## Supplementary Information

Below is the link to the electronic supplementary material.

ESM 1 Supplementary Material 1 (DOCX 1.77 MB)

## Data Availability

This study relies upon publicly available datasets which are accessible through the URLs mentioned in the text. The NCL scripts written for data analyses and visualization will be made available through a public link upon final peer-reviewed publication of the study.
