# Supplementary material for: Indo-Gangetic Plain experiences higher aerosol pollution than North China Plain despite lower emissions
Source: Environ Sci Pollut Res Int. 2026 Oct 19;33(37):17513–34. doi: 10.1007/s11356-026-38305-7 (PMC13719830; doi:10.1007/s11356-026-38305-7)
Supplement: Supplementary file 1 [file 11356_2026_38305_MOESM1_ESM.docx]

**Supplementary Material: Indo-Gangetic Plain experiences higher aerosol pollution than North China Plain despite lower emissions**

Maya Nolles^1,2^ and Tabish Ansari^1,3*^

*^1^ Campus Fryslân , University of Groningen, Leeuwarden, the Netherlands*

*^2^ The Netherlands Organisation for Applied Scientific Research, Utrecht, the Netherlands*

*^3^ Air Quality Modeling for Policy Advice, Research Institute for Sustainability at the GFZ Helmholtz Centre for Geosciences (RIFS), Berliner Str. 130, Potsdam, 14467, Germany*

*^*^ correspondence to: tabish.ansari@rifs-potsdam.de*

*Figure S1: Map of the geographical extent of the two regions analysed in this study. North China Plain (on the east coast of China) and the Indo-Gangetic Plain (in northern India) are shown in red.*
